# A pilot study of interferon-alpha-2b dose reduction in the adjuvant therapy of high-risk melanoma

**DOI:** 10.1007/s00262-019-02308-w

**Published:** 2019-02-06

**Authors:** Lorena P. Suarez-Kelly, Kala M. Levine, Thomas E. Olencki, Sara E. Martin del Campo, Elizabeth A. Streacker, Taylor R. Brooks, Volodymyr I. Karpa, Joseph Markowitz, Anissa K. Bingman, Susan M. Geyer, Kari L. Kendra, William E. Carson

**Affiliations:** 1 Comprehensive Cancer Center, The Ohio State University, Arthur G. James Cancer Hospital and Richard J. Solove Research Institute, N924 Doan Hall 410 W. 10th Ave, Columbus, OH 43210-1228 USA; 2 Medical Oncology, Department of Internal Medicine, The Ohio State University, Arthur G. James Cancer Hospital and Richard J. Solove Research Institute, Columbus, OH USA; 3 Department of Surgery, The Ohio State University Wexner Medical Center, Columbus, OH USA; 4 Promedica Toledo Hospital, Toledo, OH USA; 5 Division of Rheumatology and Center for Autoimmune Genomics and Etiology, Cincinnati Children’s Hospital Medical Center, Cincinnati, OH USA; 6 Department of Cutaneous Oncology, Moffitt Cancer Center, Tampa, FL USA; 7 Department of Biomedical Informatics, The Ohio State University College of Medicine, Columbus, OH USA; 8 Hematology, Department of Internal Medicine, The Ohio State University, Arthur G. James Cancer Hospital and Richard J. Solove Research Institute, Columbus, OH USA

## Introduction

Melanoma is a highly immunogenic tumor and consequently, efforts have been centered on the development of immune-based treatments for this malignancy [1]. Interferons were initially described in the mid-1950s as proteins that interfere with viral replication [2, 3]. Interferons are cytokines that activate Janus kinases (Jak), which lead to phosphorylation and activation of transcription factors belonging to the signal transducer and activator of transcription (STAT) family [4, 5]. Interferon-alpha (IFN-α) became available for use in clinical trials in the mid-1980s [6]. Results suggested that IFN-α inhibited the proliferation of malignant cells and stimulated immune effectors; therefore, IFN-α was initially used in patients with advanced disease [7, 8]. Since then, several meta-analyses have demonstrated that high-dose adjuvant IFN-α (daily 20 MU/m^2^ intravenous induction therapy for 1 month followed by maintenance subcutaneous 10 MU/m^2^ three times per week for at least 1 year) can prolong the disease-free interval in high-risk melanoma patients [9].

The introduction of checkpoint inhibitor therapy has revolutionized the adjuvant therapy of melanoma; however, there remains a role for IFN-α in this setting based on the potential for cancer immune escape or autoimmune events with CTLA-4 and PD-1 blocking antibodies [10–14]. There has also been significant advances in mitogen-activated protein kinase (MAPK) targeted therapies, particularly for BRAF (an intracellular signaling kinase) and MEK (signaling molecule downstream of BRAF). A recent clinical trial demonstrated significant improvement in both relapse-free survival and overall survival with adjuvant dabrafenib (BRAF inhibitor) plus trametinib (MEK inhibitor) in patients with stage III melanoma. These therapies are now approved for adjuvant therapy in BRAF mutated tumors [15]. However, since only approximately 40–50% of melanoma cells harbor an activating BRAF mutation, there still remains a role for IFN-α in this setting as the remaining 50–60% of melanomas would not be susceptible to BRAF-targeted therapies.

IFN-α activates the Jak-STAT signaling pathway and induces synthesis of hundreds of different proteins [4, 5]. Our group has shown that STAT1-mediated gene regulation within immune effectors is necessary for mediating the anti-tumor effects of IFN-α and also that the amount IFN-α administered to melanoma patients is likely in excess of the optimal biological dose [4]. Indeed, high doses of IFN-α appear to be no more effective in the induction of phosphorylated STAT1 (p-STAT1) and in the transcription of interferon-stimulated genes (ISGs) than intermediate doses [16, 17]. Our group’s studies in genetically manipulated mice have shown that suppressors of cytokine signaling-1 (SOCS1) and SOCS3 negatively regulate IFN-induced Jak-STAT signal transduction, gene regulation and anti-melanoma activity, and that high doses of IFN-α can induce SOCS proteins [18, 19].

We hypothesized that lower doses of IFN-α would be superior for induction of IFN signal transduction in patient immune cells. A prospective clinical trial was performed wherein patients eligible for adjuvant IFN-α-2b received 1 month of standard intravenous high-dose IFN-α-2b (20 MU/m^2^) followed by subcutaneous IFN-α-2b at a dose of 10 MU/m^2^ with dose reductions at set intervals down to a level of 4 MU/m^2^. Jak-STAT signal transduction and transcription of ISGs in patient peripheral blood mononuclear cells (PBMCs) were monitored during the course of adjuvant IFN-α therapy. The objective of this pilot study was to determine if lower doses of IFN-α were as effective in the induction of IFN signal transduction and gene expression as the standard high dose regimen.

## Materials and methods

### Eligibility criteria

A prospective pilot study of IFN-α-2b dose-reduction in melanoma (NCT01460875) was conducted at The Ohio State University under institutional review board approval (OSU-07033) with support from Merck Inc. Eligible patients were candidates for adjuvant IFN-α-2b after having undergone successful surgery for high-risk melanoma (Breslow thickness > 4 mm or lymph node involvement) or complete resection of metastatic disease and completion of 20 treatments of standard intravenous IFN-α-2b within 2 months of beginning treatment on this study. Patients were required to meet the following criteria: definitive surgery performed not later than 90 days prior to start of intravenous IFN-α-2b treatment, no evidence of persistent/recurrent disease, Eastern Cooperative Oncology Group (ECOG) performance status ≤ 2, life expectancy > 6 months, normal organ and marrow function, and ability to provide written informed consent.

### Treatment regimen

Prior to treatment, patients completed 20 treatments of standard intravenous IFN-α-2b (20 MU/m^2^ 5 days a week for 4 weeks). Patients then began subcutaneous IFN-α-2b injections at the standard dose of 10 MU/m^2^ thrice weekly for 4 weeks. After 1 month of therapy at 10 MU/m^2^, IFN-α-2b dose reductions were initiated. The IFN-α-2b dose was reduced to 8, 6, and 4 MU/m^2^ at 2-week intervals. The first dose of IFN-α-2b at each dose level was administered in the outpatient clinic and subsequent doses were self-administered as an outpatient. At each clinic visit, patients were evaluated for toxicities and venous blood was obtained for correlative assays. Heparinized blood samples were obtained prior to administration of IFN-α and at 1 and 4 h after administration. Once a 4-MU/m^2^ IFN-α-2b dose was achieved, patients went on to receive subcutaneous therapy for a total of 11 months. Repeat blood draws were performed every 3 months to confirm the activity of this dose.

### Clinical outcome assessment

History and physical examinations were performed every 3 months. Patients with recurrent disease were removed from trial therapy. Overall survival was defined as time to death due to any cause evaluated from time of surgery or from time of treatment initiation. Time to end of active treatment was defined as the time from start of treatment to the time patients were removed from therapy due to any cause, including completion of therapy per protocol. Patients who were event-free (e.g. alive) at their last evaluation was censored at that time point.

### Flow cytometric analysis of phosphorylated STAT1

PBMCs were isolated from patient blood via centrifugation with Ficoll-Paque Plus (Amersham Pharmacia Biotech). The phosphorylated form of STAT1 (Tyr^701^) in cryopreserved PBMCs was measured by intracellular flow cytometry, as previously described [20, 21]. Anti-p-STAT1 (Tyr^701^) conjugated antibody and isotype control antibody were obtained from BD Biosciences Pharmingen (San Jose, CA, USA).

### Reverse transcription polymerase chain reaction (RT-PCR)

Total RNA from PBMCs was extracted using Trizol reagent (Life Technologies, Grand Island, NY, USA). Reverse transcription reactions were performed using 500 ng RNA in a 20-µl reaction with the high-capacity reverse transcription kit (Life Technologies). cDNA was used as a template to measure the expression of human *SOCS1, OAS1, CXCL10*, and *CD69* genes by quantitative RT-PCR using pre-designed primers (Life Technologies). *β-Actin* served as an internal control (Life Technologies). RT-PCR reactions were performed in triplicate using the ABI PRISM 7900HT fast RT-PCR system (Applied Biosystems).

### Statistical methods

Clinical characteristics were descriptively summarized for all evaluable patients. Changes in p-STAT1 with decrease in dose levels were evaluated graphically for each patient, focusing on absolute change in p-STAT1 between dose level 10 MU/m^2^ and dose level 4 MU/m^2^ and specific p-STAT1 levels at each of these dose levels. Clinical outcomes, including toxicity and tolerability measures, were summarized across all patients, and reasons for end of active treatment were also dichotomized as completed treatment per protocol vs. not. Comparisons between groups were analyzed using Wilcoxon rank sum tests and differences between dose levels in the same patients were evaluated using the paired nonparametric Wilcoxon signed rank test. Time-to-event outcomes were analyzed graphically using the standard Kaplan–Meier methods. Modeling on time-to-event outcomes was done using average hazard ratio estimates in the Cox regression model to accommodate non-proportional hazards.

## Results

### Patient characteristics

Thirty-four patients were accrued (18 males and 16 females). Median age was 52 years (range 22–77). There was one Hispanic patient and the remainder were non-Hispanic Caucasians. The majority of the melanoma primary lesions were Clark’s level ≥ 4 and 64% were > 2.0 mm in Breslow thickness. There was regional lymph node involvement in 26 of the 34 patients, and three patients had metastases which were surgically removed. Two patients had undergone prior immunotherapy for their stage III melanoma and two had received prior radiotherapy to the primary site (Table 1).

**Table 1:** Patient and tumor characteristics

	All patients (*n* = 34)
Age (years)	
Median	52
Range	22–77
< 50	15
≥ 50	19
Gender	
Male	18
Female	16
Race	
White	33
Non-white	1
Clark’s level	
II	1
III–IV	1
IV	24
IV–V	1
V	4
Missing	3
Stage	
IIB	5
IIC	3
IIIA	8
IIIB	10
IIIC	6
IV	1
Missing	1
Lymph node involvement	
No	8
Yes	26
Primary tumor site	
Skin	33
Subdermal	1
Prior immunotherapy	
No	32
Yes	2
Prior radiation therapy	
No	31
Yes	2
Missing	1

### Adverse events and tolerability

This schedule of IFN-α-2b dosing was well tolerated by the majority of patients. The most common adverse events are listed in Table 2. Myalgia (82%), fatigue (82%), myelosuppression (79%), and mild electrolyte abnormalities (68%) were most frequently observed. Grade 3 toxicities included nausea (3%), fatigue (15%), myelosuppression (21%), elevated AST (6%), depression (6%), and electrolyte abnormalities (15%). There was one grade 4 episode of lymphopenia in a single patient. Fifteen patients (44%) completed a full year of treatment. Six patients recurred during the course of treatment. Reasons for end of active treatment are presented in Supplementary Table 1. The median time on treatment was 10.1 months (range 0.2–11.9 months).

**Table 2:** Adverse events and toxicities

	All Grades (*n* = 34)	Grade ¾ (*n* = 34)
Fever	8 (24%)	0 (0%)
Myalgia	28 (82%)	0 (0%)
Nausea	16 (47%)	1 (3%)
Vomiting	8 (24%)	0 (0%)
Fatigue	28 (82%)	5 (15%)
Myelosuppression	27 (79%)	7 (21%)
Increased AST	19 (56%)	2 (6%)
Neuropsychiatric symptoms including depression	12 (35%)	2 (6%)
Rigors/chills	19 (56%)	0 (0%)
Diarrhea	9 (26%)	0 (0%)
Electrolyte abnormalities	23 (68%)	5 (15%)
Hyperglycemia	10 (29%)	0 (0%)
Alopecia	10 (29%)	0 (0%)

*AST* aspartate aminotransferase

### Activation of STAT1 in patient PBMC

**Fig. 1:**
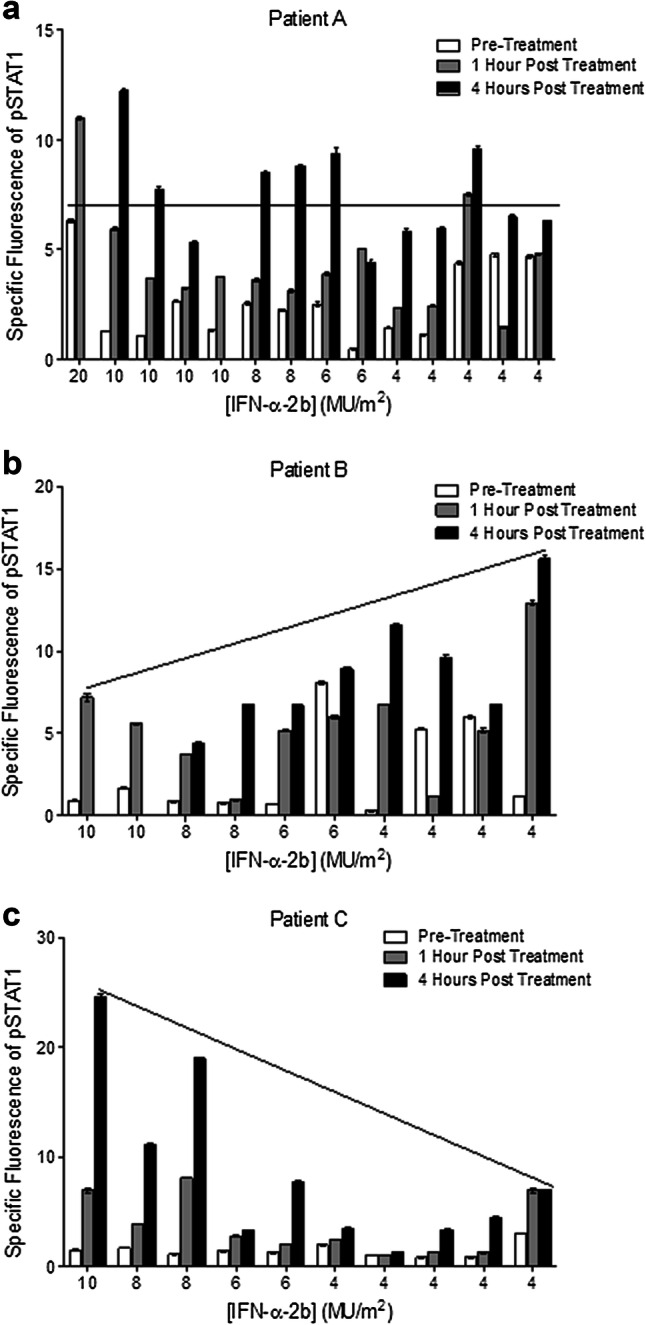
Levels of activated STAT1 following dose reduction of IFN-α-2b for three representative patients. PBMCs from 34 melanoma patients were obtained before and at 1 h and 4 h following administration of IFN-α-2b. Freshly isolated PBMCs were analyzed for p-STAT1 by flow cytometry. Isotype control antibodies were used to determine background staining. Flow cytometric data were derived from at least 10,000 events gated on the lymphocyte populations as determined by light scatter properties. Levels of activated STAT1 at different doses of IFN-α-2b for three patients demonstrate a patient who exhibited stable levels of p-STAT1 (**a**), increasing levels of p-STAT1 (**b**), or decreasing levels of p-STAT1 (**c**)

**Fig. 2:**
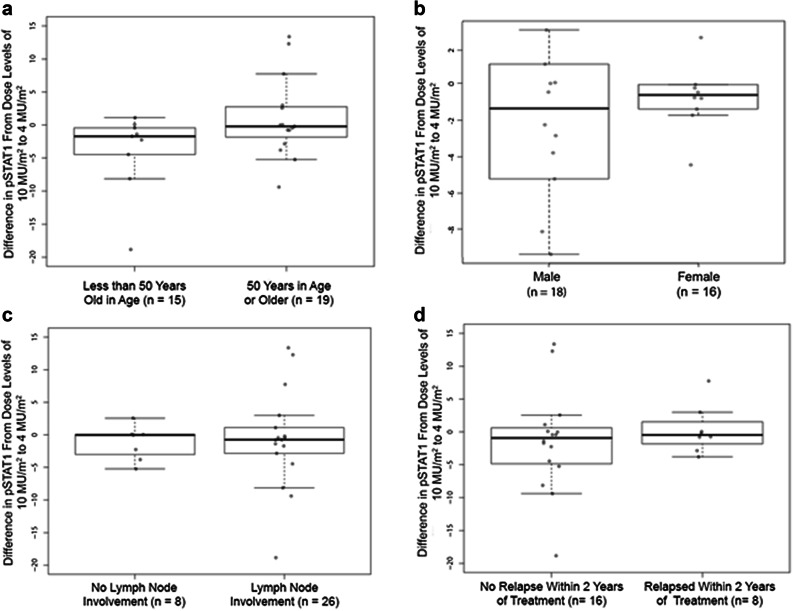
Subgroup analysis revealed no significant relationships with the levels of p-STAT1 at 10 MU/m2 vs. 4 MU/m2. Differences in levels of p-STAT1 in PBMCs following dose reduction from 10 MU/m^2^ dose to 4 MU/m^2^ dose according to age (**a**), gender (**b**), lymph node status (**c**), and relapse within 2 years of treatment initiation (**d**) are demonstrated

Levels of p-STAT1 in PBMCs were analyzed just prior to IFN-α-2b administration and at 1 h and 4 h post-administration at each dose level of IFN-α-2b. Figure 1 gives data for three representative patients depicting three potential variations to p-STAT1 levels in response to IFN-α-2b dose titration. Figure 1a is representative of patients that experienced stable levels of p-STAT1 activation. Figure 1b is representative of patients that experienced increasing levels of p-STAT1 activation. And Fig. 1c is representative of patients that experienced decreasing levels of p-STAT1 activation over the dose reduction process. Overall, this analysis revealed that in the majority of the patients (91%), the levels of p-STAT1 remained stable or actually increased over the course of dose reduction. In only 3 of the 34 patients was the level of p-STAT1 (at either the 1- or 4-h time point) at the final 4 MU/m^2^ IFN-α-2b dose lower than it was at the starting 10 MU/m^2^ IFN-α-2b dose. There was no significant difference in the levels of p-STAT1 at the 1- or 4-h time points between the 10 MU/m^2^ dose (mean specific fluorescence = 5.96, range 0.31–19.1) and the 4 MU/m^2^ dose (mean specific fluorescence = 4.80, range 0.11–21.17) for the entire patient cohort (*p* = 0.22). The level of p-STAT1 at either dose was similar regardless of age (≤ 50 years of age at the time of surgery), gender, primary tumor mitotic rate, and lymph node involvement (Fig. 2a–c, Supplementary Table 2, and data not shown). These results indicate that the higher dose of IFN-α-2b does not lead to greater activation of patient immune cells as measured by activation of STAT1.

### Overall survival (OS) and time to recurrence (TTR)

**Fig. 3:**
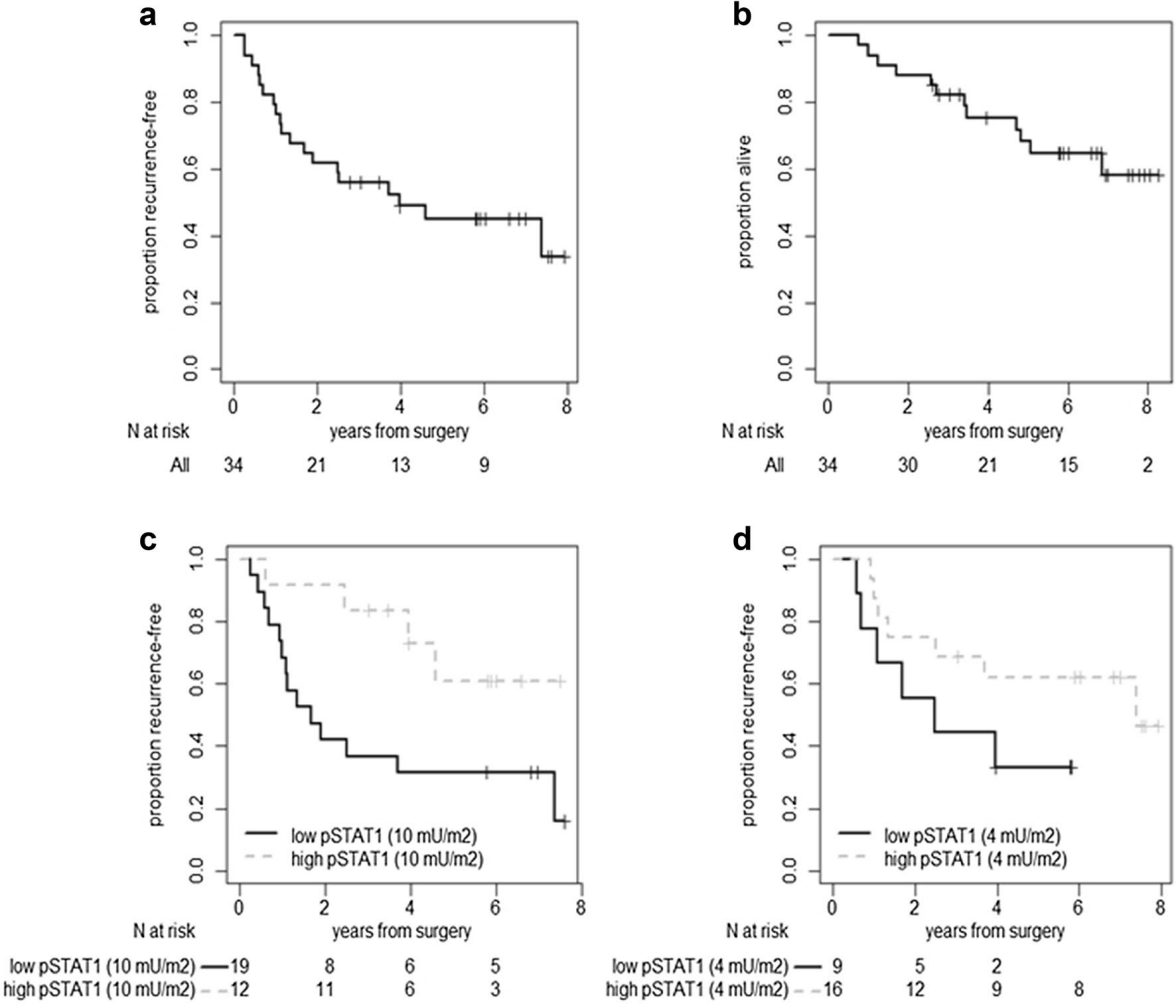
Kaplan–Meier plot of overall survival and time to recurrence. Relapse-free survival (**a**) and overall survival (**b**) are shown. An optimal cut point was determined for evaluation of the impact of high vs. low p-STAT1 expression in relation to relapse-free survival from time of treatment initiation for patients at 10 MU/m^2^ (**c**) and at 4 MU/m^2^ (**d**)

At the time of this analysis, the median follow-up for event-free subjects from time of surgery was 6.3 years (range 2.8–7.9 years) and from time of start of treatment was 6.1 years (range 2.7–7.7 years). The median time between surgery and start of treatment was 76 days (0.21 years; range 15–139 days). At last follow-up, 7 patients (21%) were alive with recurrent disease, 12 patients (35%) had died with recurrent disease, 1 patient (3%) had died without recurrent disease and a total of 14 patients (41%) were alive without recurrent disease. The median TTR from the start of treatment was 3.74 years (95% CI: 1.7 to NR) and the median TTR from the time of surgery was 3.95 years (95% CI 1.9 to NR). The 5-year estimated recurrence-free rates were the same from the time of treatment initiation and from the time of surgery: 45% (95% CI 31–66%; Fig. 3a). The median OS from time of treatment initiation and from time of surgery was 6.8 years (range: 4.7 to NR; Fig. 3b) and 6.9 years (95% CI 5.1 to NR), respectively. The estimated 5-year OS rate from time of treatment initiation was 64% (95% CI 48–82%) and from time of surgery was 66% (95% CI 52–85%).

### p-STAT1 levels and TTR

It was determined that, in addition to comparing the p-STAT1 levels after treatment with the 4 MU/m^2^ and the 10 MU/m^2^ doses, this study could also evaluate the relationship between maximal p-STAT1 levels and survival outcomes. This prompted an analysis of the relationship between survival and levels of p-STAT1 following IFN-α-2b administration. While there was no significant relationship between TTR from time of surgery or start of treatment and continuous p-STAT1 levels, we further explored if a cut-point identified through recursive partitioning for p-STAT1 at 10 MU/m^2^ and/or 4 MU/m^2^ could stratify risk of recurrence or death in these patients. We found that those patients designated as having high p-STAT1 levels (mean specific fluorescence cut-point of 6.13) after the 10 MU/m^2^ dose had a significantly lower risk of recurrence after time of surgery compared to those patients below the identified cut-point (HR = 0.31, 95% CI 0.10–0.96; *p* = 0.033; Fig. 3c). Similar results were observed when looking at time to recurrence from start of treatment (HR = 0.33, *p* = 0.044). This result suggests that a higher level of Jak-STAT signal transduction in PBMC at a dose of 10 MU/m^2^ possibly correlates with IFN-α efficacy. Although not statistically significant, patients designated as having high p-STAT1 levels (mean specific fluorescence cut-point of 2.56) after the 4 MU/m^2^ dose demonstrated lower rates of recurrence compared to those patients below the identified cut-point (HR = 0.45, *p* = 0.17; Fig. 3d).

Ten patients did not receive the 4 MU/m^2^ treatment dose, as their active treatments were stopped prior to reaching the 4 MU/m^2^ treatment dose (reasons for end of treatment are presented in Supplementary Table 1). In patients who received both the 10 MU/m^2^ and 4 MU/m^2^ doses (*n* = 24), the net difference in p-STAT1 levels between the 10 and 4 MU/m^2^ doses was not significantly associated with TTR (*p* = 0.97; HR = 1.001, 95% CI 0.93–1.08). In addition, when the data were dichotomized based on relapse within 2 years of treatment initiation, we found that there was no significant difference in the distribution of p-STAT1 between the 10 and 4 MU/m^2^ dose levels (Fig. 2d). These findings indicate that patients who had a large drop off in signaling with dose reductions from 10 to 4 MU/m^2^ did no worse than those who had a very small change in levels with the dose reduction.

### Transcription of interferon-stimulated genes (ISGs)

**Fig. 4:**
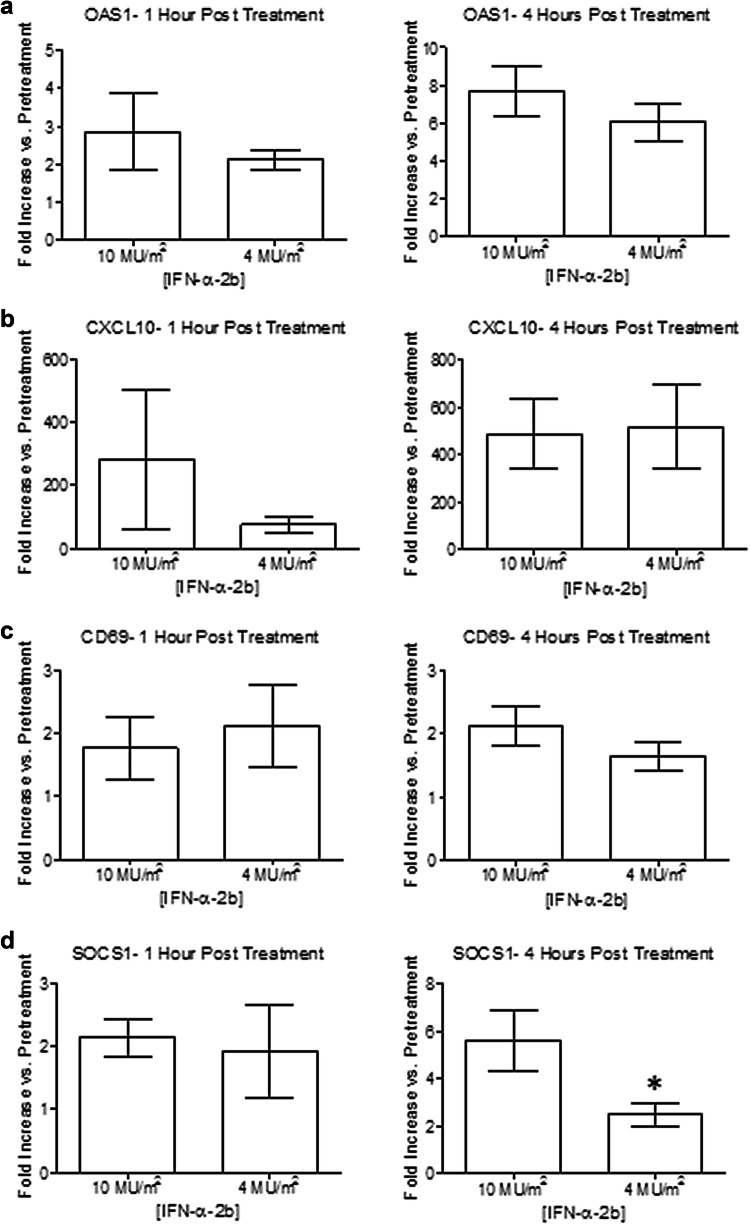
Expression of interferon-stimulated genes and SOCS1 following IFN-α-2b administration. PBMCs from 34 patients were obtained prior to IFN-α-2b treatment, 1 h post treatment and 4 h post treatment. PBMCs were lysed in Trizol reagent (Invitrogen), RNA was isolated, converted to cDNA, and analyzed by Real-Time PCR using primers specific for human OAS1 (**a**), CXCL10 (**b**), CD69 (**c**), and SOCS1 (**d**) genes. Data are expressed as the mean fold increase relative to baseline levels (pretreatment). All PCR data were normalized to the level of β-actin mRNA (housekeeping gene). Columns, mean of triplicate wells; bars, SEM (*p < 0.05 vs. 10 MU/m^2^)

Patient PBMCs were analyzed by PCR for ISGs (OAS1, CXCL10, CD69 and SOCS1) just prior to the administration of IFN-α-2b and at 1 and 4 h post-injection of IFN-α-2b. As with the levels of p-STAT1, the expression of OAS1, CXCL10, and CD69 was not significantly reduced following the reduction in IFN-α-2b dose from 10 to 4 MU/m^2^ (*p* > 0.05 for these genes; Fig. 4a–c). The expression of SOCS1 (negative regulator of Jak-STAT) was not significantly different following the reduction in IFN-α-2b dose from 10 to 4 MU/m^2^ at the 1-h post treatment time point (*p* = 0.086). However, at the 4-h post treatment time point, the 10-MU/m^2^ treatment dose induced significantly higher SOCS1 gene expression compared to the 4-MU/m^2^ treatment dose (*p* = 0.023; Fig. 4d).

## Discussion

The present study demonstrates that dose reduction of IFN-α-2b from 10 to 4 MU/m^2^ is feasible and well-tolerated. Only one grade 4 toxicity was encountered (lymphopenia) and the documented grade 3 toxicities were easily reversed and consistent with those observed in prior trials. This dose-reduction regimen was associated with estimated survival rates that were on par with the 5-year relapse-free survival (RFS) and OS rates reported in the high-dose IFN-α E1690 clinical trial (45% vs. 44% RFS and 64% vs. 52% OS, respectively) [22] and those reported in the high-dose IFN-α E1684 clinical trial (median RFS of 3.7 vs. 1.7 years and median OS of 6.8 vs. 3.8 years, respectively) [23]. Subcutaneous administration of IFN-α-2b led to the induction of p-STAT1 in circulating immune cells with no difference in the levels of p-STAT1 at the 10-MU/m^2^ dose as compared to the 4-MU/m^2^ for the group of patients as a whole. Additionally, following the reduction in IFN-α-2b dose from 10 to 4 MU/m^2^, the expression of several well-characterized ISGs (OAS1, CXCL10, and CD69) was not significantly reduced. However, the higher IFN-α-2b dose induced greater transcription of SOCS1, a negative regulator of the IFN-α immune response. In a separate analysis, higher p-STAT1 levels were found to be associated with prolonged TTR for both the 10 MU/m^2^ and 4 MU/m^2^ doses.

This pilot study provides evidence that IFN-α-2b at a dose of 4 MU/m^2^ is effective in activating circulating host immune effector cells and stimulating IFN-α induced gene expression. These results suggest that an analysis of downstream signal transduction and gene regulation may have utility in the dose selection process for IFN-α therapies. However, when looking at markers of interest to risk stratify patient populations, independent validation is required. In this study, independent validation was not performed. Therefore, data are not presented as definitive results but rather to explore and evaluate the role of p-STAT1 levels and their predictive utility in relation to clinical outcomes, such as TTR. As such, this report does not represent a true training set, but rather provides results that inform and support future larger studies evaluating the role of p-STAT1 levels in relation to clinical outcomes. Similarly, with respect to p-STAT1 level cutoff points, these results need to be validated and verified in larger, confirmatory studies before being applied in clinical practice.

Multiple IFN-α regimens have been evaluated as adjuvant therapy for intermediate or high-risk melanomas, with mixed results. The Eastern Cooperative Oncology Group (ECOG) trials E1684 and E1694 demonstrated durable improvement in both RFS and OS utilizing a high-dose IFN-α regimens [23, 24]. However, the E1690 high-dose IFN-α trial only demonstrated durable improvement in RFS [22] and the Sunbelt Trial demonstrate no RFS or OS benefit with the use of high-dose IFN-α [25]. Given the toxicity of high-dose IFN-α, several clinical trials have evaluates low- and intermediate-dose regimens with, again, mixed results. While evaluating the utilization of intermediate-doses of adjuvant IFN-α, the European Organization for Research and Treatment of Cancer (EORTC) 18991 and the Nordic IFN trials demonstrated improvement in RFS, [26, 27], whereas the EORTC 18952 trial demonstrated no RFS or OS benefit [28]. However, on subgroup analysis the EORTC 18952 trial did demonstrate a durable improvement in RFS and OS in patients with ulcerated melanomas [28].

Likewise, several clinical trials have been performed to evaluate the efficacy of low-dose adjuvant IFN-α in high-risk melanoma patients [22, 29–36]. The Dermatologic Cooperative Oncology Group (DeCOG) trial demonstrated durable improvement in both RFS and OS [29], while the French Cooperative Group trial demonstrated significant extension of RFS and a clear trend towards increased overall survival (*p* = 0.059) [30]. The Italian Skin Cancer Foundation, Austrian Malignant Melanoma Cooperative Group, and Scottish Melanoma Group clinical trials demonstrated significant improvement in RFS, but no improvement in OS [31–33]. However, no improvement in either RFS or OS was seen in the WHO Melanoma Programme, AIM HIGH Study-United Kingdom Coordinating Committee on Cancer Research, EORTC 18871, or ECOG 1690 trials [22, 34–36]. The vast heterogeneity of treatment regimens used and mixed treatment responses seen within these trials makes interpretation of the results difficult. Most studies have failed to define clinical or demographic features that would identify patients more likely to respond to IFN-α treatment regimens. However, since STAT1-mediated gene regulation within immune effector cells is necessary for mediating the anti-tumor effects of IFN-α [4], the variations in treatment responses seen in these studies may be the result of the varied ability of the administered low-dose IFN-α to activate the Jak-STAT signaling pathway on a patient-by-patient basis. Therefore, individual evaluation of Jak-STAT signaling in response to IFN-α treatment may be able to identify patients that are more likely to benefit from adjuvant treatment and aid in dose optimization.

The use of phosphorylation-state specific antibodies for intracellular flow cytometry has a unique potential for the evaluation of signaling events in immune effectors following the administration of immunomodulatory cytokines. Until the precise molecular determinants of IFN-α-responsiveness are identified, it seems reasonable to use signal transduction within immune effector cells as a surrogate marker of IFN-α action in patients undergoing immunotherapy. The present trial only evaluated the response of each patient to a pre-determined schedule of dose reduction. Given the inter-patient variability in p-STAT1 induction to a given dose of IFN-α, it is likely that the optimal dose of cytokine (i.e., that dose which induces the greatest activation of immune cells as measured by the induction of p-STAT1) would be different for each patient. We, therefore, anticipate that this method might be useful as a means of identifying the dose of IFN-α which produces optimal Jak-STAT signal transduction on a patient-by-patient basis.

A previous clinical trial of patients with metastatic melanoma evaluated the treatment regimen of bevacizumab in combination with escalating doses of IFN-α-2b (5 MU/m^2^ for 2 weeks and then 10 MU/m^2^ thereafter) [37]. Levels of p-STAT1 at a dose of 5 MU/m^2^ IFN-α-2b were greater or equivalent to those at a dose of 10 MU/m^2^ for six of the seven patients studied. Similarly, the induction of ISGs within PBMCs at a dose of 5 MU/m^2^ was greater or statistically equivalent to that observed for 10 MU/m^2^ IFN-α-2b for six of the seven of the patients. Microarray analysis was performed on five patients with metastatic melanoma undergoing immunotherapy with escalating doses of IFN-α-2b. Analysis of the gene expression profile within PBMCs from these patients revealed that a total of 35 genes (e.g., CD69, CXCR6, IL8, PBEF) were induced to a greater extent with 5 MU/m^2^ as compared to 10 MU/m^2^ IFN-α-2b (fold induction ≥ 2). Notably, SOCS1 and SOCS3 transcripts were significantly higher in patient PBMC following the 10-MU/m^2^ dose of IFN-α-2b. These results, along with the results presented in this study, suggest that lower doses of IFN-α-2b may be just as effective as higher doses with respect to the induction of Jak-STAT signal transduction and ISG expression within immune effector cells.

There have been significant advancements in the adjuvant therapy of melanoma with the use of checkpoint inhibitors and targeted therapies for BRAF mutant melanomas. However, there still remains a role for adjuvant IFN-α in the setting of BRAF wild-type melanomas and in patients at increased risk of cancer immune escape or autoimmune events with CTLA-4 and PD-1 blocking antibodies. Although high-dose IFN-α is approved for the adjuvant treatment of melanoma, the substantial treatment-related toxicities have impeded the adoption of this regimen. The present study demonstrates that IFN-α-2b dose reduction is feasible, well tolerated, and associated with reasonable response rates. Additionally, this study provides data to support the contention that the standard subcutaneous dose of IFN-α may be higher than is necessary for maximal activation of the immune system. This study offers a novel method for potential dose-optimization, on a patient-by-patient basis, through individual analyses of signal transduction and gene regulation. However, the clinical impact of dose-optimized adjuvant IFN-α in patients with high-risk melanoma on OS and RFS needs to be evaluated in a large, randomized controlled trial.

### Electronic supplementary material

Below is the link to the electronic supplementary material.

Supplementary material 1 (PDF 28 KB)

## Abbreviations

ECOG: Eastern Cooperative Oncology Group
EORTC: European Organization for Research and Treatment of Cancer
DeCOG: Dermatologic Cooperative Oncology Group
IFN-α: Interferon-alpha
ISG: Interferon stimulated gene
Jak: Janus kinases
MAPK: Mitogen-activated protein kinase
OS: Overall survival
PBMCs: Peripheral blood mononuclear cells
p-STAT1: Phosphorylated STAT1
RFS: Relapse-free survival
SOCS: Suppressors of cytokine signaling
STAT: Signal transducer and activator of transcription
TTR: Time to recurrence

## References

[1] Cutaneous M, DeVita V, Hellman S, Rosenberg S (2001). Principles and practice of oncology.

[2] Isaacs A, Lindenmann J (1957). Virus interference. I. The interferon. Proc R Soc Lond B Biol Sci.

[3] Raaijmakers MIG, Rozati S, Goldinger SM, Widmer DS, Dummer R, Levesque MP (2013). Melanoma immunotherapy: historical precedents, recent successes and future prospects. Immunotherapy.

[4] Lesinski GB, Anghelina M, Zimmerer J, Bakalakos T, Badgwell B, Parihar R, Hu Y, Becknell B, Abood G, Chaudhury AR, Magro C, Durbin J, Carson WE (2003). The antitumor effects of IFN-alpha are abrogated in a STAT1-deficient mouse. J Clin Investig.

[5] Sen GC (2001). Viruses and interferons. Annu Rev Microbiol.

[6] Dorr R (1993). Interferon-alpha in malignant and viral diseases: a review. Drugs.

[7] Bart RS, Porzio NR, Kopf AW, Vilcek JT, Cheng EH, Farcet Y (1980). Inhibition of growth of B16 murine malignant melanoma by exogenous interferon. Can Res.

[8] Gresser I, De Maeyer-Guignard J, Tovey MG, De Maeyer E (1979). Electrophoretically pure mouse interferon exerts multiple biologic effects. Proc Natl Acad Sci USA.

[9] Davar D, Kirkwood JM (2016). Adjuvant therapy of melanoma. Cancer Treat Res.

[10] Spain L, Larkin J (2016). Weighing up the pros and cons of immune checkpoint inhibitors in the treatment of melanoma. Immunotherapy.

[11] Orloff M, Ryan W, Matias EV, Takami S (2016). Immune check point inhibitors combination in melanoma: worth the toxicity?. Rev Recent Clin Trials.

[12] Fecher LA, Agarwala SS, Hodi FS, Weber JS (2013). Ipilimumab and its toxicities: a multidisciplinary approach. Oncologist.

[13] Weber JS, D’Angelo SP, Minor D, Hodi FS, Gutzmer R, Neyns B, Hoeller C, Khushalani NI, Miller WH, Lao CD, Linette GP, Thomas L, Lorigan P, Grossmann KF, Hassel JC, Maio M, Sznol M, Ascierto PA, Mohr P, Chmielowski B, Bryce A, Svane IM, Grob J-J, Krackhardt AM, Horak C, Lambert A, Yang AS, Larkin J (2015). Nivolumab versus chemotherapy in patients with advanced melanoma who progressed after anti-CTLA-4 treatment (CheckMate 037): a randomised, controlled, open-label, phase 3 trial. Lancet Oncol.

[14] Larkin J, Chiarion-Sileni V, Gonzalez R, Grob JJ, Cowey CL, Lao CD, Schadendorf D, Dummer R, Smylie M, Rutkowski P, Ferrucci PF, Hill A, Wagstaff J, Carlino MS, Haanen JB, Maio M, Marquez-Rodas I, McArthur GA, Ascierto PA, Long GV, Callahan MK, Postow MA, Grossmann K, Sznol M, Dreno B, Bastholt L, Yang A, Rollin LM, Horak C, Hodi FS, Wolchok JD (2015). Combined nivolumab and ipilimumab or monotherapy in untreated melanoma. N Engl J Med.

[15] Long GV, Hauschild A, Santinami M, Atkinson V, Mandalà M, Chiarion-Sileni V, Larkin J, Nyakas M, Dutriaux C, Haydon A, Robert C, Mortier L, Schachter J, Schadendorf D, Lesimple T, Plummer R, Ji R, Zhang P, Mookerjee B, Legos J, Kefford R, Dummer R, Kirkwood JM (2017). Adjuvant dabrafenib plus trametinib in stage III BRAF-mutated melanoma. N Engl J Med.

[16] Lesinski GB, Kondadasula SV, Crespin T, Shen L, Kendra K, Walker M, Carson WE (2004). Multiparametric flow cytometric analysis of inter-patient variation in STAT1 phosphorylation following interferon Alfa immunotherapy. J Natl Cancer Inst.

[17] Zimmerer JM, Lehman AM, Ruppert AS, Noble CW, Olencki T, Walker MJ, Kendra K, Carson WE (2008). IFN-alpha-2b-induced signal transduction and gene regulation in patient peripheral blood mononuclear cells is not enhanced by a dose increase from 5 to 10 megaunits/m^2^. Clin Cancer Res.

[18] Lesinski GB, Badgwell B, Zimmerer J, Crespin T, Hu Y, Abood G, Carson WE (2004). IL-12 pretreatments enhance IFN-alpha-induced Janus kinase-STAT signaling and potentiate the antitumor effects of IFN-α in a murine model of malignant melanoma. J Immunol (Baltimore, Md: 1950).

[19] Alexander WS (2002). Suppressors of cytokine signalling (SOCS) in the immune system. Nat Rev Immunol.

[20] Kirkwood JM, Bender C, Agarwala S, Tarhini A, Shipe-Spotloe J, Smelko B, Donnelly S, Stover L (2002). Mechanisms and management of toxicities associated with high-dose interferon alfa-2b therapy. J Clin Oncol.

[21] Glaspy JA (2002). Therapeutic options in the management of renal cell carcinoma. Semin Oncol.

[22] Kirkwood JM, Ibrahim JG, Sondak VK, Richards J, Flaherty LE, Ernstoff MS, Smith TJ, Rao U, Steele M, Blum RH (2000). High- and low-dose interferon Alfa-2b in high-risk melanoma: first analysis of intergroup trial E1690/S9111/C9190. J Clin Oncol.

[23] Kirkwood JM, Strawderman MH, Ernstoff MS, Smith TJ, Borden EC, Blum RH (1996). Interferon alfa-2b adjuvant therapy of high-risk resected cutaneous melanoma: the Eastern Cooperative Oncology Group Trial EST 1684. J Clin Oncol.

[24] Kirkwood JM, Ibrahim JG, Sosman JA, Sondak VK, Agarwala SS, Ernstoff MS, Rao U (2001). High-dose interferon Alfa-2b significantly prolongs relapse-free and overall survival compared with the GM2-KLH/QS-21 vaccine in patients with resected stage IIB-III melanoma: results of intergroup trial E1694/S9512/C509801. J Clin Oncol.

[25] McMasters KM, Egger ME, Edwards MJ, Ross MI, Reintgen DS, Noyes RD, II RCGM, Goydos JS, Beitsch PD, Urist MM, Ariyan S, Sussman JJ, Davidson BS, Gershenwald JE, Hagendoorn LJ, Stromberg AJ, Scoggins CR (2016). Final results of the sunbelt melanoma trial: a multi-institutional prospective randomized phase III study evaluating the role of adjuvant high-dose interferon Alfa-2b and completion lymph node dissection for patients staged by sentinel lymph node biopsy. J Clin Oncol.

[26] Eggermont AMM, Suciu S, Santinami M, Testori A, Kruit WHJ, Marsden J, Punt CJA, Salès F, Gore M, MacKie R, Kusic Z, Dummer R, Hauschild A, Musat E, Spatz A, Keilholz U (2008). Adjuvant therapy with pegylated interferon alfa-2b versus observation alone in resected stage III melanoma: final results of EORTC 18991, a randomised phase III trial. Lancet.

[27] Hansson J, Aamdal S, Bastholt L, Brandberg Y, Hernberg M, Nilsson B, Stierner U, von der Maase H (2011). Two different durations of adjuvant therapy with intermediate-dose interferon alfa-2b in patients with high-risk melanoma (Nordic IFN trial): a randomised phase 3 trial. Lancet Oncol.

[28] Eggermont AMM, Suciu S, Rutkowski P, Kruit WH, Punt CJ, Dummer R, Salès F, Keilholz U, de Schaetzen G, Testori A (2016). Long term follow up of the EORTC 18952 trial of adjuvant therapy in resected stage IIB–III cutaneous melanoma patients comparing intermediate doses of interferon-alpha-2b (IFN) with observation: ulceration of primary is key determinant for IFN-sensitivity. Eur J Cancer.

[29] Garbe C, Radny P, Linse R, Dummer R, Gutzmer R, Ulrich J, Stadler R, Weichenthal M, Eigentler TK, Ellwanger U, Hauschild A (2008). Adjuvant low-dose interferon α2a with or without dacarbazine compared with surgery alone: a prospective-randomized phase III DeCOG trial in melanoma patients with regional lymph node metastasis. Ann Oncol.

[30] Grob JJ, Dreno B, de la Salmoniere P, Delaunay M, Cupissol D, Guillot B, Souteyrand P, Sassolas B, Cesarini J-P, Lionnet S, Lok C, Chastang C, Bonerandi JJ (1998). Randomised trial of interferon α-2a as adjuvant therapy in resected primary melanoma thicker than 1·5 mm without clinically detectable node metastases. Lancet.

[31] Rusciani L, Petraglia S, Alotto M, Calvieri S, Vezzoni G (1997). Postsurgical adjuvant therapy for melanoma. Cancer.

[32] Pehamberger H, Soyer HP, Steiner A, Kofler R, Binder M, Mischer P, Pachinger W, Auböck J, Fritsch P, Kerl H, Wolff K (1998). Adjuvant interferon alfa-2a treatment in resected primary stage II cutaneous melanoma. Aust Malig Melanoma Cooper Group.

[33] Cameron DA, Cornbleet MC, Mackie RM, Hunter JAA, Gore M, Hancock B, Smyth JF (2001). Adjuvant interferon alpha 2b in high risk melanoma—the Scottish study. Br J Cancer.

[34] Cascinelli N, Belli F, MacKie RM, Santinami M, Bufalino R, Morabito A (2001). Effect of long-term adjuvant therapy with interferon alpha-2a in patients with regional node metastases from cutaneous melanoma: a randomised trial. Lancet.

[35] Hancock BW, Wheatley K, Harris S, Ives N, Harrison G, Horsman JM, Middleton MR, Thatcher N, Lorigan PC, Marsden JR, Burrows L, Gore M (2004). Adjuvant interferon in high-risk melanoma: the AIM HIGH Study—United Kingdom coordinating committee on cancer research randomized study of adjuvant low-dose extended-duration interferon Alfa-2a in high-risk resected malignant melanoma. J Clin Oncol.

[36] Kleeberg UR, Suciu S, Bröcker EB, Ruiter DJ, Chartier C, Liénard D, Marsden J, Schadendorf D, Eggermont AMM (2004). Final results of the EORTC 18871/DKG 80-1 randomised phase III trial: rIFN-α2b versus rIFN-γ versus ISCADOR M® versus observation after surgery in melanoma patients with either high-risk primary (thickness > 3 mm) or regional lymph node metastasis. Eur J Cancer.

[37] Zimmerer JM, Lehman AM, Ruppert AS, Noble CW, Olencki T, Walker MJ, Kendra K, Carson WE (2008). IFN-α-2b-induced signal transduction and gene regulation in patient peripheral blood mononuclear cells is not enhanced by a dose increase from 5 to 10 megaunits/m^2^. Clin Cancer Res.

