# Supplementary material for: A pilot study of interferon-alpha-2b dose reduction in the adjuvant therapy of high-risk melanoma
Source: Cancer Immunol Immunother. 2019 Feb 6;68(4):619–29. doi: 10.1007/s00262-019-02308-w (PMC6447692; doi:10.1007/s00262-019-02308-w)
Supplement: Supplementary file 1 — Supplementary material 1 (PDF 28 KB) [file 262_2019_2308_MOESM1_ESM.pdf]

Supplementary Table 1. Off-Study Reasons

	All Patients (n = 34)
Adverse events/Complications	5 (15%)
Disease progression/Relapse	6 (17%)
Withdrawal or Refusal	5 (15%)
Completed per Protocol	15 (44%)
Other	3 (9%)

Supplementary Table 2. Influence of Patient Characteristics on IFN-α-2b Response

		pSTAT1 Difference Dose 4 MU/m^2 – Dose 10 MU/m^2 median (range)	p-value
Age (years)			
	< 50	-1.71 (-18.84 – 1.11)	0.14
	$\geq$ 50	-0.20 (-9.38 – 13.36)	
Gender			
	Male	-1.34 (-18.84 – 13.36)	0.67
	Female	-0.60 (-4.45 – 7.74)	
Lymph Node Involvement			
	No	-0.02 (-5.22 – 2.57)	0.95
	Yes	-0.74 (-18.84 – 13.36)	
